# Insects as Source of Nutraceuticals with Antioxidant, Antihypertensive, and Antidiabetic Properties: Focus on the Species Approved in Europe up to 2024

**DOI:** 10.3390/foods14081383

**Published:** 2025-04-17

**Authors:** Annalaura Brai, Claudia Pasqualini, Federica Poggialini, Chiara Vagaggini, Elena Dreassi

**Affiliations:** Department of Biotechnologies, Chemistry and Pharmacy, University of Siena, Via Aldo Moro 2, I-53100 Siena, Italy; pasqualini5@student.unisi.it (C.P.); federic.poggialini@unisi.it (F.P.); chiara.vagaggini@student.unisi.it (C.V.); elena.dreassi@unisi.it (E.D.)

**Keywords:** edible insects, *Acheta domesticus*, *Alphitobus diaperinus*, *Locusta migratoria*, *Tenebrio molitor*, antihypertensive, functional food

## Abstract

Insects represent a traditional food in different parts of the world, where eating insects is not only related to nutrition, but also results from a variety of sociocultural customs. Insects’ nutritional profiles typically vary by species. Nevertheless, in terms of nutrition, edible insects can be a rich source of protein, dietary fiber, healthy fatty acids, and micronutrients, including minerals and vitamins. Insects have a low carbon footprint and require fewer resources in terms of land, water, and food with respect to animal livestock. Interestingly, insects are a source of bioactive compounds with different pharmacological activities, including antioxidant, antimicrobial, antidiabetic, antiobesity, antihypertensive, and antilipidemic. Among the bioactive compounds, polyphenols, chitosan, and protein hydrolysates are the most important ones, with direct activity on ROS quenching and enzymatic inhibition. Glucosidase, DPP-IV, ACE, and lipases are directly inhibited by insects’ bioactive peptides. Lipids and tocopherols reduce inflammation and lipid peroxidation by acting on LOX and COX-2 enzymes and on ROS quenching. The insects’ nutrient composition, coupled with their easy and economical breeding, is the cause of the growing interest in edible insects. During the last 20 years, the study and development of novel insect-based products increased, with relevant effects on the market. This review focuses on the edible insects currently approved in Europe, namely, *Acheta domesticus*, *Alphitobus diaperinus*, *Locusta migratoria*, and *Tenebrio molitor*. The nutrient profile and the functional compounds are examined, with an eye on market trends and on the patent applications filed in the last decades.

## 1. Introduction

Insects are the largest arthropod class, with more than 1 million species described; among them, more than 2100 are edible [1]. Most of the edible species have been eaten for centuries in Africa, Asia, Oceania, or Latin America, and are thus perfectly safe for human consumption. Even if entomophagy is part of the traditional culinary culture of billions of people worldwide, eating insects is uncommon and often not acceptable for many people in the EU countries [2,3]. This phenomenon, known as entomophobia, is probably related to the novelty represented by the introduction of novel foods, as already experienced for tomatoes in the 1500s and sushi in the early 1900s. The market size for insect protein has been estimated at more than 235 million worldwide in 2023, and it is expected to grow at a compound annual growth rate of 28.9% in the next ten years [4]. According to recent reports, the European market for edible insects is projected to reach 2.98 billion by 2032 [5]. The EU market growth is probably related to the increasing interest in climate change and to the favorable opinion of scientists and institutions about insects [6]. The major consumers are the Netherlands, Germany, the UK, France, and Belgium; nevertheless, the animal nutrition segment has the highest market share. According to the Forbes data, in Germany only the market had an annual growth of 24% in the last six years [7]. The key companies are Ÿnsect (SAS) (Évry-Courcouronnes, France), Protix B.V. (S Hertogenbosch, The Netherlands), InnovaFeed SAS (Paris, France), EntoCube OY (Espoo, Finland), Cricket Lab Limited (London, UK), and BIOFLYTECH S.L. (Fuente Alamo de Murcia, Spain), while other small and medium-sized enterprises are being established.

According to recent reports, there are notable variations between the countries. Even if the insect consumption is high in the regions that traditionally consume insects, more people in Portugal, Poland, Slovenia, and Spain are inclined to try insects. Curiosity or a shortage of food were determined to be the main drivers of insect consumption [8]. The major concerns are related to the safety of edible insects, in particular the presence of allergens and contamination with persistent organic pollutants, mycotoxins, or microorganisms [9]. Accordingly, novel industrial processing technologies are rapidly expanding, with an eye on the reduction of waste and the development of novel products from by-products [10].

Population growth is one of the challenges of our time. As reported by the Food and Agriculture Organization (FAO) [11], millions of people experience food insecurity and malnutrition due to the cost of healthy diets. Furthermore, it is impossible to produce an adequate quantity of proteins with current farming methods due to the low sustainability of the process. In 2013, the FAO pointed out the potential application of insects as food and feed, highlighting the advantages and pitfalls of this resource [12]. Insects are considered a complete and healthy food because they have a balanced amount of nutrients. The protein content is similar to fish but different from vegetables, comprising all of the essential amino acids in the recommended ratios [13]. Interestingly, insects have high-quality fat with short-chain fatty acids amounting to less than 40%, and some of them are also a valuable source of micronutrients such as iron and vitamin B12. The nutrient concentration differs based on the species. The most interesting characteristic is the quantity of micronutrients, in particular, minerals and vitamins, that can be exploited in controlled regimens. Analyzing feed conversion efficiency, insects have lower land and hydric requirements [14,15,16] and water pollution and greenhouse gas emissions in comparison to conventional livestock [17]. Furthermore, higher fecundity, less susceptibility to diseases and zoonosis [18], and higher feed conversion efficiency are other factors contributing to their increasing popularity [16,19]. Recent studies demonstrate that insects can be reared in a multiplicity of by-products, upcycling them in the productive stream. Insects are also advantageous in terms of emissions, producing less greenhouse gases (GHGs) and ammonia than mammals [20]. These characteristics make them suitable as protein sources, also for developing countries [21]. Nowadays, the food demand is increasing, and so is the need for a more sustainable and equally distributed food supply chain [22].

The aim of a circular bioeconomy prompts the research for alternative and innovative food sources to protect both the climate and the environment [23]. According to this vision, edible insects have received particular attention in recent years [24] as a promising alternative to meet the increasing food demand while providing high nutritional value [25]. Despite the practice of eating insects being ancient, entomophagy is currently in the spotlight for its potential as a novel and sustainable food system [26].

As reported in a recent survey by IPIFF, consumer acceptance should be increased thanks to the nutraceutical substances of insects [27]. In fact, edible insects have an important antioxidant activity, which is related to their functional properties. Among the bioactive compounds, there are chitosan and a large number of peptides with proven antihypertensive and antimicrobial properties [28]. Another important advantage is the antiobesity effect of chitosan, which, coupled with the increased level of satiety of certain species, can represent a valuable solution for controlled regimens. Preliminary results showed that the effects on satiety are species-specific and depend on the quantity of insect flour ingested.

According to the European regulation EU 2015/2283, novel foods for human consumption can be marketed only after a positive evaluation by the European Food Safety and Authority (EFSA). Accordingly, two species of insects have been approved in 2021, *Tenebrio molitor* larvae (TML) and *Locusta migratoria* (LM) [29,30]. In early 2023, two other species were approved by the EFSA: house crickets (AD) (*Acheta domesticus*) and the larvae of *Alphitobius diaperinus* [31,32]. This review focuses on the edible insects currently approved in Europe, namely, *Acheta domesticus, Alphitobus diaperinus, Locusta migratoria,* and *Tenebrio molitor*. The nutrient profile and the nutraceuticals are examined, with an eye on market trends and on the patent applications filed in the last decades.

## 2. Edible Insects Approved in Europe

*Tenebrio molitor* (TM), also known as yellow mealworm, is a species of darkling beetle of the family *Tenebrionidae*, order Coleoptera (Phylum Arthropoda, Class Insecta) [33]. As the Latin etymology of the name suggests, these insects prefer dark environments and are common storage pests [34], mainly distributed in the northern temperate regions [35]. TM is a holometabolic insect, and its life cycle goes through four different development stages, egg, larva, pupa, and adult [36], characterized by significant changes in the mealworm morphologies [37]. Environmental conditions such as diet, temperature, relative humidity, darkness, and population density are crucial points for the developmental stages of TM [14,15,16,38]. TM can be reared in very simple conditions by untrained personnel, using different diets (wheat bran and enrichment with organic matter, i.e., potatoes, carrots, apples) and limited space [39]. Tropical regions have a long tradition of entomophagy [40], and this insect is consumed at the larval stage in many areas of Africa, Asia, and Latin America [41,42,43,44]. Despite insect consumption still encountering barriers in Western countries, according to the EFSA [45], TM belongs to the list of insects with the highest potential as food and feed; TM larvae (TML) were the first insect-based novel food authorized [46] by EFSA in 2021. Specifically, EFSA stated that the consumption of TML in the form of powder or as dried insects used as ingredients in various food products does not raise any safety concerns and has a low allergenic risk.

The term “migratory locust” refers to the adult stage of the insect species *Locusta migratoria*, which is a member of the genus *Locusta* and subfamily *Locustinae* of the family *Acridae* [47]. These insects represent the most widespread grasshopper species in the world and are currently present in various regions, including Australia, Asia, Africa, and Europe [29]. LM exists in two phenotypes, solitary and gregarious, determined by the environment [48]. These insects are consumed in 65 countries, especially in Africa and Asia, and they are prepared in different ways, including boiling, toasting, roasting, frying, and sun-drying. Due to their short life cycles, locusts can reach adulthood in four to eight weeks, depending on their rearing conditions. Since they may be raised vertically, less land is needed for their upkeep. The nutritional and chemical composition of *Locusta migratoria* suggests that it can be a healthy food source for people and a viable treatment to ward off a variety of illnesses.

In 2018, the company Fair Insects BV (a Protix Company) submitted a request to the European Commission to authorize placing migratory locusts on the market as a novel food in frozen form without legs and wings, dried form without legs and wings, or powder form with legs and wings. Removal of wings and legs in frozen and dried formulations allows for reducing intestinal constipation brought on by ingesting the big spines on the insect’s tibia. Fair Insects BV proposed to use this novel food as an ingredient in various food products such as breakfast cereals, pasta, bakery products, sauces, meat products, and meat imitates. Products with one of the migratory locusts’ formulations could be consumed by the general population. After careful study of the submitted data regarding the nutritional content by the applicant, the EFSA authorized the placing of the migratory locust reared and harvested during the solitary phase as novel food on the market. On 25 May 2021, the EFSA panel stated the safety of frozen and dried formulations from migratory locusts, including potato and legume-based dishes, pizza, meat imitates, soups, salads, chocolate, pasta, snacks, beer and beer-like beverages, alcoholic drinks, and liqueurs [29].

*Acheta domesticus* (genus *Acheta*, family *Gryllidae*, order *Orthoptera*, suborder *Ensifera*) is the most commonly farmed cricket. Over 2400 cricket species have been identified worldwide, with nearly half of them belonging to the *Gryllidae* family. The *Gryllidae* family contains 62 popular edible species, the majority of which are found in Asia (41 species) and Africa (26 species), with 5 species found in America, 4 in Europe, and 4 in Australia. AD is the most commonly reared cricket species for human consumption [49]. The use of AD as a novel food has been approved by the EC. EU Regulation 2022/188 approved the commercialization of AD in frozen, dry, and powder form, while EU Regulation 2023/5 approved the marketing of AD in partially defatted powder form. The two regulations’ ratification resulted in a modification of the previously issued EU rule 2017/2470. Despite not being listed in EU 2017/2470, AD has been amended and added to the new food list based on EU 2023/5. AD is considered safe as a food ingredient in crackers, breadsticks, meat imitates, and snacks. AD exhibited no cytotoxicity in three human cell types (HL60, HeLa, and Caco-2). Even though no report on the toxicity and genotoxicity of AD has been published, EFSA has expressed concerns about biological and chemical hazards [50].

*Alphitobius diaperinus* (Panzer, 1797) is a *Coleoptera* member of *the Tenebrionidae* family. Its larval form is often called lesser mealworm (LMW). LMW originates from sub-Saharan Africa, but it is diffused worldwide. LMW was so far considered a pest of grains or henneries, as well as a vector for avian pathogens like *Salmonella* and *Escherichia coli* [51]. For these reasons, considerable efforts have been dedicated to the search for effective treatments to eradicate this insect. Its reproductive cycle consists of an initial egg stage, followed by 6 to 11 larval instars, a prepupa stage, a pupa stage, and the final adult stage. Its growth is facilitated by humidity and warm conditions, and temperature can reduce the time between instars, and influence the pupal rate and weight [52]. The maximum larval length is 7 to 11 mm during the last instar. Adults can live up to 12 months and lay over 2000 eggs [53,54]. The EFSA panel declared the use of frozen and freeze-dried formulations of LMW whole or in the form of a paste or powder safe. Nevertheless, the panel highlights the potential risk of allergies. The Commission Implementing Regulation (EU) 2023/58 of 5 January 2023 authorizes the placing of frozen, paste, dried, and powder forms of LMW on the market as a novel food.

## 3. Nutrient Profile

Insects are an important source of proteins, essential amino acids, fats, minerals, vitamins, and bioactive substances [18,55,56]. Knowledge of nutritive values, health benefits, and safety procedures is essential for promoting edible insects’ consumption.

Table 1 summarizes the proximate composition of the approved edible insects reported by EFSA and the literature [38,43,57,58,59,60,61] fed with standard diets.

According to the reported data, all of the approved insects are mainly composed of proteins. AD has the highest protein content, followed by LMW, TML, and LM. The fat content ranges from 37 to 10% and is high in LM, followed by TML, LMW, and AD. Carbs ranged from 4.62% in crickets to 0.82% in LM, and fibers ranged from 8 in AD to 5.66% in TML.

TML have a high content of proteins and high-quality lipids. Proteins represent the main component of the nutrient composition of TML. However, the quality and value of the protein profile must be assessed through the amino acid content [58]. In addition to the highly valuable protein profile, TML are also interesting as a source of lipids, with an average lipid content of 30–35% of dry weight (Table 1). According to the excellent nutritional profile, it is no surprise that TML have been recommended as a bioregenerative life support system for space missions [41,62]. Fiber content in TML (typically around 5%) is dependent on the different compounds bound to chitin, a key component of edible insects’ exoskeleton [63]. Quantifying crude protein content in insects should consider the high chitin level of a non-protein nitrogen (NPN) polysaccharide found in the exoskeleton of insects, which could overestimate the protein levels [64]. Chitin, a polymer of β-(1–4)-N-acetyl-D-glucosamine, is an undigestible fiber with protective effects on human health such as hypocholesterolemic [65], haemostatic [66,67], and immunomodulatory effects [68]. From a nutritional point of view, TML have a protein content lower than that of beef and chicken, but equal to or higher than that of lamb and pig [69]. Fat content is lower than that of lamb and pork, but it is higher than that of beef and chicken. The FAO and WHO advise a 75 kg man to consume between 2550 and 4000 kcal per day, depending on his basal metabolic rate, to fulfil his energy demands [70]. Athletes need a higher energy intake and a different nutritional consumption level. While proteins can reach up to 2.2 g/kg of body weight, carbohydrates should be raised to a maximum of 12 g/kg of body weight. Due to the higher energy requirements, the suggested fat amount is between 20 and 35 percent of total fat consumption [70,71,72]. Considering those numbers, TML can meet the fat and protein needs of both athletes and the general public, while carbohydrates fall short of the required range often seen for meats [73].

As for TML, LM are noted for their high protein content, with averages around 50 and 60% [74]. Carbohydrates, of which chitin is a part, constitute a low percentage compared to other nutrients varying from 4 to 6%. Fat constitutes the second main component of the LM body. Triglycerides constitute 80% of the insect’s body, while phospholipids are less than 20%. When measured on a dry weight basis, LM have a fat quantity of 13%. Considering other meats, LM have a protein content higher than lamb but comparable to pork and lower than beef and chicken. Fat content is lower than lamb and pork but higher than beef and chicken. If we consider fish, the protein content is lower, but the fat is comparable to salmon and higher than cod, tuna, and rainbow trout [75,76]. Considering the energy requirements, LM have a high energy rate value of 545 kcal/100 g, higher than cod and tuna, but lower than meats. Depending on their nutrition, LM have different nutrient compositions. The insect’s body fat content increased when carrots or wheat bran were added to their diet; however, wheat bran lowered the protein amount [77]. Additionally, carrots did not affect the composition of α and β-carotene concentration; instead, wheat bran decreased carotene concentration. The amount of vitamin A in migratory locusts’ bodies increased when wheat bran and carrots were added to their diet.

Proteins are the most abundant nutrient in crickets. It has been demonstrated that the protein content ranges between 55 and 75% of dry matter. The variation is caused by diets and methodology, most importantly, the conversion factor used in the Kjeldahl assay. AD also had low fat content when compared to other edible insects (10.4% of dry matter), but higher protein (71.7% of dry matter). The fat content of AD matched that of *orthoptera* species. Among polysaccharides, chitosan was isolated from AD trapped in lipids. The lipid-binding capacity is 168.7–210.8 g oil per g of chitosan, comparable to shrimp chitosan, which exerts antiobesity activity [78]. The protein value is comparable to chicken and higher than lamb, pork, and salmon [75]. Lipid content is about two times higher than in lean fishes like cod and tuna, but lower than in salmon, rainbow trout, and meats. Low fat content makes these crickets appealing to food industries due to their higher shelf-life and applications in sport nutrition.

As for the other insects, LMW are mainly composed of proteins (58–65%), followed by fats (13–29%), carbs (3%), and fibers (6–7%). Notably, the dry matter is lower than the other approved insects, except for AD. The protein content is comparable to TML and higher than lamb and pork. The diet and the conversion factor used for protein determination are the most impactful factors in protein values [64]. As for TML, the fat is higher than beef and fish like cod, tuna, and rainbow trout, but lower than lamb and pork [75]. The energy values are about 460 kcal/100 g DW, a value slightly below those of common meats, but able to satisfy FAO and WHO daily requirements [11,79].

Significant variations are reported between the already published data. Interestingly, the differences about the individual content of macronutrients are mainly related to the diets and to the rearing conditions [80]. The poor comparability of data can be due to the absence of a bromatological analysis of the feeds and to the poorly described rearing conditions, including developmental stage, relative humidity, and temperature [81,82,83].

As reported in Table 2, insects are rich in fats in quantities ranging from 10 to 37% of DM. Regarding FA composition, the most abundant saturated fatty acid is palmitic acid, ranging from 19 to 28%. The most abundant mono and polyunsaturated fatty acids are oleic and linoleic acids. Notably, MUFAs represent the most abundant FA in TML and LMW, while PUFAs are the most abundant FA in AD. Omega-3 and omega-6 fatty acids such as α-linolenic and α-linoleic acids are essential PUFAs because humans cannot synthesize them, and therefore must be outsourced from the diet [84]. The *n*6/*n*3 ratio is outside the recommended range in TML, AD, and LMW, while it is below 2 in LM [85]. Lowering the ratio between omega-6 and omega-3 (*n*6/*n*3 ratio) is important for controlling the risk for inflammation, carcinogenesis, and cardiovascular diseases, with optimal values being around 1–4 [86,87]. Despite the *n*6/*n*3 ratio in almost all the insects approved being high due to the abundance of α-linoleic acid, it must be observed that the fatty acid composition of the insects can exhibit great variability and is highly dependent on the insect feed [88]. Specifically, a diet with low contents of omega-3 acids could explain the high *n*6/*n*3 ratio. Conversely, it has been reported that a diet supplemented with omega-3 fatty acid sources such as linseed enhances the omega-3 content in TML, thus reducing the *n*6/*n*3 ratio to more suitable values [88,89,90]. The nutritional indices associated with cardiovascular pathology prevention are comparable to those of seaweeds, fish, and chicken [91].

As presented in Table 3, the content of amino acids may vary among the literature, depending on the diets, origins, and developmental stage, but also differences in the techniques employed for the determination and the nitrogen-protein conversion factor adopted [43,57]. Nevertheless, the cited studies indicate that insects are both “rich in” protein and an alternative “source of” protein [62]. Noteworthy, insects provide amounts of the required essential amino acids (EAA) close to the values recommended by the FAO [92]. Table 3 highlights that TML possess a high content of Val, Leu, and Lys [40,43,57,58,59,92,93], and can represent a significant source of sulphur-containing amino acids, important for their anticancer, anti-inflammatory, and antioxidant properties [94,95,96]. LM has a lower content of sulphur and aromatic amino acids [97,98], but even in this case fulfils all of the EAA suggested by the FAO per gram of protein. Among the unessential amino acids, Gly and Ala are significantly higher with respect to the other insects.

AD have an important content of sulphur amino acids, as well as aromatic amino acids [99]. AD contains all essential amino acids in an amount comparable to those of egg, chicken, pork, and beef, which are considered the primary protein sources in the human diet. Particularly interesting is the high lysine and threonine contents, which could help supplement cereal-based diets, which are generally low in these essential amino acids. Additionally, arginine was found in high concentrations. In humans, arginine is considered an essential amino acid because synthesis is insufficient for growth and development in children. The total quantity of essential amino acids is slightly below the values observed for TML. As observed for TML, LMW fulfil all of the FAO/WHO EAA requirements. Both sulphur and aromatic amino acids are abundant [64,100]. These insects can thus represent a valid alternative for consumers looking for alternatives to conventional meat sources [77,101,102].

As reported in Table 4, numerous micronutrients have been shown to vary significantly between and within species. This probably represents the real variance in micronutrient concentration caused by seasonality, nutrition, and geographic location, especially in wild insects. Variations in the micronutrient makeup of farmed insects may be explained by feed composition. Insects have a valuable mineral composition [40], and TML are no exception [55,103], being rich in minerals like iron, zinc, copper, and magnesium (Table 4), essential for many metabolic and physiological processes [104,105,106,107,108]. In general, the approved insects can fulfill the suggested minerals’ daily requirements. The mineral content of TML is comparable to meats like pork and lamb, and in some cases, higher than seafood products [75,109]. It is undeniable that these insects can improve the micronutrient-poor diets common in developing countries, contributing mostly to iron and zinc deficiencies [40,58].

The contents of some major minerals (Na, Mg, P, K, and Ca) and trace minerals (Mn, Fe, Cu, and Zn) in cricket species differed. The mineral contents of AD were demonstrated to be generally higher than those of other edible crickets. The results revealed that phosphorus was the most abundant, followed by potassium, calcium, magnesium, and sodium. Phosphorus is required for ATP and nucleic acid synthesis (RNA and DNA), as well as protein production. Calcium and magnesium are essential components of bones. The calcium concentration of the crickets was higher than that of milk (90–130 mg/100 g), implying that crickets could be used as an alternative source of calcium. It is worth noting that the synthesis of haemoglobin depends on iron. According to the mineral composition, crickets have the potential to be good sources of dietary minerals [110,111].

Basically, the mineral content of 100 g of dry LM ranges from 8 to 100 mg. In comparison to poultry, beef, and pork, these edible insects possess an equal amount of zinc and a higher ratio of iron measured in mg/100 g dry matter [112]. According to Table 4, LM has calcium levels that are equal to or higher than those of beef and pork, which range from 4 to 28 mg/100 g [75]. It has an equivalent or greater concentration of the essential micronutrients iron and zinc than beef and pork, which had levels of 1–6 and 2.4–12.5 mg/100 g, respectively. As with other meals like fruits and vegetables, the amounts of heavy metals in LM are within recognized limits, and their zinc content is similar to that of beef and pork [75,113].

LMW have a mineral content ranging from 1.9 to 200 mg per 100 g of insect [114]. LMW are abundant in important elements like calcium and iron [76]. The major mineral component is sodium, followed by magnesium, potassium, zinc, iron, and copper. The iron content is higher than in fish like cod, haddock, and shrimp, but comparable to lamb and beef. The content of zinc is higher than in seafood, in particular fish and crustaceans like shrimp and lobster, but slightly below pork and comparable to chicken, lamb, and beef [75,109].

## 4. Use of Insects as Functional Foods and Sources of Bioactive Compounds

Bioactive compounds are extra nutritional constituents of food present in small quantities that produce positive effects on human health. They generally include PUFAs, peptides, polyphenols, carotenoids, and vitamins [115,116]. Insects are an interesting source of bioactive peptides, lipids, and sterols [56,103,117]. Despite the literature about vitamins being limited, studies have indicated that edible insects are also interesting for their vitamin composition, pointing out that it is highly dependent on the species, the soil, and the feeding diet [23]. By comparing available data, Nowak et al. [55] found that TML can be a good source of vitamin B6 (pyridoxine), B2 (riboflavin), B3 (niacin), B9 (folate), and B12 (cobalamin), as well as vitamin D3 (cholecalciferol) [117]. LM also contains retinol, vitamin D, vitamin B12, a variety of carotenoids [118,119], and antioxidant peptides that can guard against conditions like diabetes, cardiovascular disease, skin issues, chronic kidney, neurodegenerative disorders, and so forth [120]. Additionally, it was discovered that LM dry matter has antioxidant action against oxidative stress through the capacity to chelate metal ions, with copper ions having the maximum chelating capability [121]. 

Table 5 offers a schematic overview of the most important bioactive compounds found in approved edible insects and their biological activity. Among bioactive peptides, antihypertensive TML peptides have raised particular interest. Dai et al. [122] were the first to observe an angiotensin-converting enzyme (ACE)-inhibitory tripeptide, YAN, obtained from TML hydrolysate (IC_50_ = 172.1 μM), and demonstrated its activity in spontaneously hypertensive rats. Brai et al. identified three other ACE-inhibitory peptides, NIKY (IC_50_ = 52.2 μM), QGLGY (IC_50_ = 264 μM), and HILG (IC_50_ = 2.82 mM), and also investigated their binding mode, providing the in vitro evidence that the antihypertensive activity of TML peptides is probably due to a cumulative effect derived from the presence of several antihypertensive compounds [19]. Several peptides with ACE-inhibitory activity were also found in LM hydrolyzed proteins, among them were TCDSL, IDCSR, and EAEEGQF [120]. Due to their inhibition of ACE, both TML and LM should be included in a standard diet to prevent hypertension and reduce the use of inhibitory drugs. These findings pave the way for the use of EFSA-approved insects as potential ingredients in functional foods intended for blood pressure regulation.

Other peptide fractions comprise compounds implied in blood sugar regulation, and thus may represent a novel strategy for the food-based control of type II diabetes, another important red flag of the metabolic syndrome. Specifically, two important enzymes are α-glucosidase, which hydrolyses complex sugars into digestible monosaccharides in the intestine [151], and DPP IV, implied in the regulation of incretins half-life, important peptides for the regulation of blood sugar level [125,126,127,152].

Zielinska et al. [140] identified two major peptides with α-glucosidase inhibitory activity and showed that heat treatment of the protein hydrolysates not only conserved their effect, but also improved it. Rivero-Pino et al. [141] identified some peptide fractions (from 500 to 1600 Da) with promising DPP IV inhibitory activity, and other fractions (below 500 Da) correlated with α-glucosidase in vitro inhibition. Based on the molecular features, some peptides from TML and AD were suggested to be the bioactive compounds responsible for the described inhibitions (e.g., APVAH for DPP IV inhibition, CSR for α-glucosidase inhibition) [125,126,127,152]. Edible crickets have antioxidant, anti-inflammatory, antidiabetic, and antiobesity properties, which enhance the positive effects of their consumption [126,127].

Chen et al. [142] identified two promising peptides endowed with antithrombotic activity and studied their binding mode with molecular modeling techniques, highlighting interaction with thrombin 1 exosite. The cardioprotective activity of TML lipids was further proved in a mouse model.

With the threat of antimicrobial resistance always increasing [153], the discovery of novel antimicrobial agents is of utmost importance. Antibacterial and antifungal properties have also been observed in edible insects’ proteins in different in vitro studies. Tenecin 1 is an inducible protein of the defensin family secreted in TML hemolymph active against Gram-positive bacteria [154]. Tenecin 2 and Tenecin 4 [145] are inducible coleoptericin/diptericin-like and attacin/gloverin-like molecules, respectively, which mainly target Gram-negative bacteria. Tenecin 3 is a constitutive [155] glycine-rich peptide with antifungal activity, provoking the lysis of fungi-infected cells [146].

Chitin is another bioactive compound of edible insects that can be converted into chitosan by deacetylation. This polymer is widely applied in environmental, food, and biomedical areas [115], and recent studies have highlighted its antibacterial and anti-inflammatory properties [129,130,136,137]. In addition, chitosan has important antidiabetic, antihyperlipidemic, and antiobesity properties, as reported in different clinical trials [138,156]. In light of the vast applicability of chitin and chitosan, whose global market increased with an annual growth rate of 15.4% from 2016 to 2023 [157], insects and their exuviae can be an attractive source of these compounds.

Oxidative stress is a pathological condition resulting from an imbalance between the production and removal of ROS and consequent pro-inflammatory molecule generation, resulting in cellular and tissue damage. This is ultimately correlated with metabolic syndrome, diabetes, cardiovascular disease, ageing, cancer, and neurodegenerative processes [158]. Other important compounds with antioxidant activity are polyphenols. Due to insects’ capacity to accumulate compounds from feed, different agricultural, marine, and food industry by-products have been used to increase the antioxidant activity [73,131,132,133,134,159]. The upcycling strategy has multiple advantages: (a) the insects increase the mean weight and speed up their growth; (b) they increase the content of nutraceuticals with antioxidant activity; and (c) they increase the product shelf life.

Adult AD extracted using ultrasound-assisted extraction (UAE) with absolute ethanol or 50% (*v*/*v*) aqueous ethanol exhibited antioxidant activity. The antioxidant and anti-inflammatory effects of edible insects are due partly to their MUFA and PUFA content, but also to some peptide hydrolysates [147] that act as iron-chelating agents and 5-LOX/COX-2 inhibitors, as studied in vitro by Zielinska et al. [147].

Furthermore, the ROS-scavenging properties of insects’ peptides make them promising candidates against ROS-induced hepatotoxicity. Research conducted by Cho et al. [148] demonstrated the hepatoprotective effect of TML alkaline hydrolysate and identified two hepatoprotective peptides, AKKHKE and LE. The cytoprotective effect against H_2_O_2_-induced toxicity in AML12 mouse cells is due to increases in NF-E2-related factor 2-mediated expression of catalase, heme-oxygenase 1, and glutathione synthesis-related genes [148].

Bioactive lipids comprise the already described MUFAs and PUFAs (Table 2) and tocopherols such as γ-tocopherol [150], all contributing to the aforementioned antioxidant and anti-inflammatory properties. Among phytosterols, stigmasterol and β-sitosterol were identified, known for their hypocholesterolemic effects [117].

Despite the extraction and recovery of bioactive compounds needing robust studies, the nutraceutical and pharmaceutical potential of insects’ peptides and lipids is evident in both in vitro and animal studies. These findings strongly encourage their use in nutraceutical formulations both as food and feed. It is fundamental to highlight that many endogenous compounds, like large polypeptides and polyphenols, are poorly adsorbed in vivo. In addition, even if the presence of polyphenols facilitates the quenching of ROS, proper storage conditions should be used to maintain their effectiveness and prevent their oxidation before reaching the target.

## 5. Applications in Plastic Degradation and Circular Economy

Polystyrene (PS) is one of the major sources of plastic products and one of the most persistent environmental pollutants, contributing to ocean microplastics [160]. Biodegradation is one of the hottest topics of environmental research to fight the massive environmental contamination [161], and TML were the first insect larvae capable of degrading petroleum-based plastic polymers [162].

Studies demonstrated that PS undergoes depolymerization within the TML gut, converting ingested PS into CO_2_ and biomass with a total carbon recovery efficiency above 95%, highlighting the TM capability of degrading PS [162]. Interestingly, the mealworm gut is an effective bioreactor enabling PS biodegradation, due to PS-degrading bacteria [163], similarly to lignocellulose breakdown in ruminant mammals. The ability of TML to digest PS can be defined as ubiquitous and independent from the geographic origin of the mealworms, since it has been described in 22 countries [164], confirming that mealworms from different locations can metabolize PS. Yang et al. further demonstrated TML-based polyethylene (PE) degradation, suggesting a non-specific degradation pattern and how the mealworm gut microbiome can adapt to chemically different plastic polymers [165]. Polystyrene seems to also be metabolized by LMW, thanks to the diversity of intestinal microorganisms [166]. A recent study also highlighted the utilization of polyurethane polymers (PU) by TML [167]. To the best of our knowledge, the safety of the use of plastic-degrading insects for food or feed purposes has not investigated.

Nowadays, the model of a linear economy based on the “take, make, use, and waste” dogma is no longer applicable due to socioeconomic problems, environmental crises, and climate change [168]. Circular economy (CE) [169] is a model of production and consumption based on the restorative industrial system of the “grow-make-use-restore” approach in order to reduce waste to a minimum. It has been previously outlined that insect rearing and entomophagy are very promising from a circular economy standpoint. Furthermore, in addition to their potential as an alternative source of both food and feed, TM are efficient biomass converters [170].

Insects can transform organic waste into nutritious feedstuff by converting organic waste matter to protein, thus contributing significantly to the circular production system [171]. The waste is reintroduced into the food production chain, according to the CE principles [172]. As the farming of insects becomes more popular in Europe and the United States, the use of food industry by-products in insect feed is gaining attention. However, with current production technology, it is still being determined how to maximize the use of by-products in feeds while meeting nutritional needs and preserving successful rearing yields. By-products can be used in insect feeds because many species can feed on an array of plant materials, in place of conventional feed ingredients. Several studies have already demonstrated that by-products from the food industry and agriculture can be used to make suitable feed ingredients for farmed insects [50]. By-products of the agricultural industry enriched in proteins could be used as an alternative to the traditional source of proteins in insect feeds, but they meet the nutritional needs of the insects only as a feed supplement [131,132,133,173,174,175]. Sorjonen et al. assessed the growth performance of the most widely used edible cricket specie, *A. domesticus*, on 18 experimental diets [176]. The diets included commercial chicken feeds and cricket diets in which soybean was partially or completely replaced with food industry by-products such as potato protein, barley mash, barley feed, compressed leftover turnip rape, and a mix of broad bean and pea on three protein levels. The high- and medium-protein turnip rape and barley mash diets yielded the highest yield and improved all performance variables. Overall, high- and medium-protein diets resulted in the highest yield, growth, and development. Their findings indicated that food industry by-products could be used in cricket feeds, furthering the goals of the circular economy. Protein by-products have significant potential as feed for insects, including AD. Many of these diets promote greater growth, development, and yield when compared to control diets. However, diets primarily composed of organic waste or by-products (low-value diets) may result in lower cricket growth performance and survival. This suggests that diets consisting solely of by-products may be deficient in important, nutritionally necessary components for cricket development and growth. However, their research shows that when other nutritional components, such as carbohydrates and fats, are balanced in the diet, by-products can be used as a protein source for crickets [176].

In this context of “zero-waste”, the frass derived from edible insects is another interesting insect component to exploit and capitalize on [177]. Due to its content of nitrogen, phosphorus, and potassium (NPK) and its rapid mineralization, it is a valid substitute for conventional NPK mineral fertilizers [178]. Frass of TML, AD, and LM have been investigated as potential fertilizers [179]. The frass is also an excellent material for biochar production, a renewable carbon source involved in wastewater purification of heavy metals [180]. A recent study also investigated the utilization of TM frass-based biochar for CO_2_ capture technology and supercapacitor materials [181].

Finally, due to their fat content, TM and LMW are studied for their role in biorefinery processes and biodiesel production [182]. Despite more trials and investigations being needed, all these examples demonstrate the enormous potential of edible insects in circular food systems.

## 6. Market Analysis of Edible Insects

The global market for edible insects has raised considerable interest in recent years due to the high nutritional and protein value of insect-based products, combined with their low sugar and caloric content [56].

Despite skepticism, food neophobia, and the disgust factor still being great hurdles to insect consumption in Western countries [183], the edible insect global market is forecasted to reach USD 9.14 billion by 2034, at a Compounded Average Growth Rate (CAGR) of 28.3% during 2024–2034 [184]. The insects-as-food market is expected to grow faster than the insects-as-feed market, and among the product types (such as whole insects, insect powder, insect meal, and insect oil), insect powder is the product expected to grow at the fastest CAGR in 2024–2034. In the panorama of novel insect foods, TML and AD are considered the main references [184].

Concerning the typology of products traded, the whole insect is the most representative product (50% of the market scene), followed by insect meal (20%). Insect meals differ in their insect content and can be divided into three main categories: products above 90% insect content, products between 10% and 90% content, and products with less than 10% insect content [185]. The latter type is mostly found among protein bars, snacks, and pasta [184], which are specifically designed to attract larger groups of customers without the disadvantage of strong insect visibility, which can trigger phobia and repulsion [186].

According to a study by Orsi et al. [183], sociodemographic factors impact the attitude towards entomophagy, with younger people being more positively interested. In this regard, appealing packaging and attention to the insect-based food’s visual aspects and nutritional content are crucial to attract the target group of customers. Cricket farms and industries in Western countries formulate various types of cricket-based foods in the form of dried cricket and flour, such as cricket bars, to serve Western populations that are unfamiliar with entomophagy [111].

According to a survey from Pippinato et al. [185], the market supply is still managed by a small group of companies, mostly from Northern Europe (Great Britain, Denmark, Finland, and France), with e-commerce being the main distribution channel in Europe. Most companies sell processed raw materials, while only 20% of them (12 out of 59 companies) are also rearing the insects and thus produce their own raw materials.

Regulation (EU) 2015/2283 (EFSA Scientific Committee 2015) now applies to insect-based products as part of the EU’s new regulatory framework on the production and marketing of novel foods. Due to the complexity of this regulation, the placing on the market of novel insect products requires following the EFSA authorization procedure. On one hand, the times related to the authorization are long and may be prohibitive for small and medium enterprises, but on the other hand, they are necessary to guarantee the consumers’ safety. The reduction of the time associated can represent a win-win strategy for both the EU and insect producers. As for the other meals, insect producers are required to follow the hazard analysis and critical control point (HACCP) rules, which represent another important guarantee for the consumer.

According to a recent IPIFF survey, the safety of the novel products and the nutraceutical compounds of insects should boost customer consumption. For this purpose, some functional qualities of edible insects should be exploited to encourage their consumption. The antioxidant activity is fundamental to preventing age-related pathologies, while chitosan and bioactive peptides, with antihypertensive and antibacterial qualities, are among the most important bioactive substances. Chitosan’s antiobesity action is another significant benefit, especially if combined with some species’ capability to increase the level of satiety [78,85,128].

Novel products like cookies and pasta are emerging, in addition to the energy bars. The use of edible insects added as a powder to well-known dishes is aimed at increasing consumer acceptance and reducing the disgust sensation, which still represents a critical barrier.

TM-based product average prices in the European market are generally linked to the insect quantity, with the highest value for whole insect TM products (22.1 EUR/100 g), followed by insect meals (10.7 EUR/100 g) and the lowest prices for protein bars (2.0 EUR/100 g). Moreover, sales format also plays a role in determining the price, showing that smaller formats have the highest price per 100 g [185].

## 7. Patents

The growing interest in edible insects is also confirmed by the increasing number of documents and patents published over the years 2010–2024 [115,187]. Table 6 provides an overview of the most relevant patents and patent applications published worldwide since 2010. Regarding the applicants, the majority of applications came from China, followed by Korea, the USA, and EU countries. The majority of patent applications refer to the commercial use of TML, followed by AD, LMW, and LM. Interestingly, the applications cover the use of edible insects as food and feed, including rearing procedures. Due to the presence of many bioactive compounds, several applications are related to the procedure of active component extraction, as well as to the nutraceutical use for pathologies and as cosmetic ingredients. An increasing number of applications are related to the use of insects as plastic degraders.

## 8. Safety Concerns

Insects reared in accordance with food safety regulations are considered safe to eat. Nevertheless, as for the other foods, they need to be analyzed for the presence of contaminants, including persistent organic pollutants, heavy metals, mycotoxins, and pathogenic microorganisms like viruses and bacteria [1,188,189].

The potency of allergens present in insect proteins can vary depending on processing methods. While some studies demonstrated the ineffectiveness of such treatments on the immunoreactivity of insect proteins found in house crickets [190,191], others found that the immunoreactivity of migratory locusts was eliminated when exposed to extreme heat treatments or enzymatic hydrolysis [192]. In addition, the allergenicity can be modified based on the product consumed, due to the presence of many different ingredients.

The mycotoxins of Fusarium, Aspergillus, and Penicillium may also be found in an insect’s gut. This suggests that there may be associated food safety concerns because these toxins may have both acute and long-term impacts on both humans and animals. In addition, different quantities of mycotoxins have also been discovered in approved edible insects [193].

The accumulation of pesticides and dioxins is another possible contamination. Even if some insects like TML could degrade some pesticides like epoxiconazole, further studies are required to test individual persistent organic compounds [194]. On the contrary, Camenzuli demonstrated that heavy metals could accumulate with the insects, based on the concentration of the substance in the feeding substrate, on the individual species, and on the growth stage [193]. Another weakness in the supply chain for edible insects has been found to be the processing of insects, which is common in EU countries, to mask the aspect of insects. Thanks to the heat treatment and drying procedures, the overall number of microorganisms is reduced with respect to the fresh weight [195,196]. Future studies should be dedicated to traceability of insects in processed foods. HPLC and NMR multivariate analyses already offered different tips to better understand the food composition and discriminate between commercial products.

## 9. Conclusions

Currently, four different edible insects have been approved in Europe for human consumption: *Acheta domesticus*, *Alphitobus diaperinus*, *Locusta migratoria*, and *Tenebrio molitor*. The growing interest in edible insects is a result of their nutrient composition, as well as their low carbon footprint and simple and affordable breeding process. Insects are rich in digestible proteins, and have a high quantity of MUFA and PUFA, which make them particularly useful for athletes and people under controlled regimens for cardiovascular diseases. Interestingly, several studies highlight that edible insects are rich in bioactive compounds, including peptides with antimicrobial and antifungal activity, formed in the human body during gastrointestinal digestion. Different patent applications are related to the pharmaceutical exploitation of bioactive compounds, which have in many cases complementary activities. Diabetes and hypertension are common comorbidities, which have similar risk factors, including obesity, vascular inflammation, and dyslipidemia. Notably, insects exert antidiabetic properties, inhibiting α-glucosidase and DPP-IV enzymes, but also have an important antihypertensive activity, due to the presence of different inhibitory peptides which inhibit the ACE enzyme. In addition, tocopherols compete with cholesterol for absorption, limiting their plasmatic concentration. Finally, the presence of antioxidants with ROS quenching effects and high-quality FA further promotes the use of insects as functional foods. Taken together, the functional properties of insects are important to prevent and limit cardiovascular diseases, largely diffused in developed countries.

Even if entomophobia is still diffused in Europe, due to the novelty represented by the introduction of novel foods, the market is growing fast with a CAGR of 28.3% during 2024–2034. Future efforts should be addressed to prove their potential as a functional food, and promote their consumption. As demonstrated by the IPIFF survey, consumers are more motivated to eat foods rich in nutraceuticals, due the wellness-associated benefits and the possibility to reduce the development of age-related pathologies. Finally, lessons learned from novel foods like tomatoes in the 1500s and sushi in the early 1900s lead us to believe that their diffusion is just around the corner.

## Figures and Tables

**Table 1:** Analysis of the proximate composition of edible insects reared on standard diets. Results are reported in g per 100 g of insect #.

	Crude Protein		Fat		Total Digestible Carbs		Dietary Fiber		Ash		Moisture		Chitin	
	Mean	SD	Mean	SD	Mean	SD	Mean	SD	Mean	SD	Mean	SD	Mean	SD
LMW frozen	17.71	4.07	8.21	0.81	0.82	0.22	1.42	0.16	1.11	0.04	69.30	1.43	Nr	Nr
LM frozen	13.90	1.28	11.28	0.82	0.22	0.04	2.58	0.08	0.78	0.11	71.50	0.00	1.77	0.02
LMW paste	15.91	4.66	6.06	0.69	Nr	Nr	2.22	0.28	0.92	0.06	72.30	1.37	2.20	0.19
AD powder	64.35	26.95	10.38	1.36	4.62	1.08	8.84	0.66	5.00	0.56	4.82	1.25	6.26	1.64
LMW powder	55.60	14.93	23.88	1.58	2.65	0.46	6.86	0.38	3.53	0.10	2.76	1.15	Nr	Nr
LM powder	51.77	7.99	35.63	2.49	2.08	0.31	7.02	0.48	1.90	0.00	1.38	0.58	11.72	0.69
LM dried	46.82	4.28	37.98	2.73	0.82	0.04	8.70	0.26	2.62	0.41	4.28	0.08	6.50	0.07
LMW dried	53.93	15.74	25.27	1.59	Nr	Nr	6.54	0.37	3.59	0.09	2.37	0.38	7.84	0.62
TML dried	53.37	12.39	26.28	4.18	3.58	2.96	5.66	1.18	4.00	0.27	2.58	2.00	6.42	0.28

# Results comprehend edible insects in selected forms approved by EFSA [29,30,31,32]. *Acheta domesticus* (AD); *Alphitobius diaperinus* (LMW); *Locusta migratoria* (LM); *Tenebrio molitor larvae* (TML). Nr: not reported.

**Table 2:** Analysis of the fatty acids and nutritional indices associated with cardiovascular pathologies in edible insects reared on standard diets. Results are reported in g per 100 g of total fatty acids ^#^.

FA	TML	LM	AD	LMW
C14:0	3.57	1.67	Tr	Tr
C16:0	18.62	28.44	24.26	24.29
C16:1	1.32	Tr	Tr	Tr
C18:0	5.33	8.27	9.19	8.7
C18:1	35.63	31.6	23.3	37.16
C18:2	28.23	16.96	36.67	22.57
C18:3n − 3	1.18	10.47	2.55	Tr
C24:1	1.08	Tr	Tr	Tr
SFA	31.17	39.21	35.18	36.41
MUFA	38.42	33.02	25.06	39.21
PUFA	30.41	27.77	39.76	24.38
PUFA/SFA	0.98	0.71	1.13	0.67
n−3	1.49	10.67	2.75	1.15
n−6	28.77	16.97	37.01	23.09
n−6/n−3	19.43	1.59	13.47	20.16
IA	0.48	0.58	0.41	0.44
IT	0.72	0.67	0.86	0.98
H/H	2.94	1.96	2.54	2.44

^#^ Results of edible insects approved by EFSA [73,74,75,76,77,78,79,80]. *Acheta domesticus* (AD); *Alphitobus diaperinus* (LMW); *Locusta migratoria* (LM); *Tenebrio molitor larvae* (TML). Tr: values < 0.4; SFA: saturated fatty acid; MUFA: monounsaturated fatty acid; PUFA: polyunsaturated fatty acid.

**Table 3:** Analysis of amino acids in EFSA-approved edible insects. Results are reported in g per 100 g of proteins ^#^ [98].

Amino Acid	TML	LM	AD	LMW	FAO/WHO (2013) Standard [92]
Met	1.52–1.75	0.07–1.42	1.50–2.2	1.59–1.71	1.6
Cys + cys	1-10–1.15	0.87–4.40	0.85–1.46	0.84–0.96	
Total sulphur AA	2.9	2.29	3.65	2.56	
Tyr	5.80–8.11	5.14–5.64	4.74–6.26	6.16–8.49	3.8
Phe	3.64–4.39	3.23–3.89	2.20–4.07	4.22–5.17	
Total aromatic AA	12.49	8.87	10.33	10.38	
Ile	4.94–4.99	4.61–4.66	3.60–4.46	4.61–4.77	3
Leu	7.88–8.33	8.35–8.44	6.31–7.79	7.32–7.36	5.9
Lys	5.2–6.14	5.01–5.72	5.2–5.80	7.057.35	4.5
Thr	4.28–4.52	3.82–3.96	3.1–4.07	4.31–4.42	2.3
Val	6.42–7.13	6.46–7.1	5.20–6.59	5.76–6.4	3.9
His	3.55	2.72–2.87	2.92–3.39	3.21–3.97	1.5
Total EAA	48.37	43.06	45.01	46.44	
Ser	5.03–5.05	4.07–4.08	3.71–5.36	4.41–4.66	
Arg	5.57–5.94	5.91–6.2	5.73–7.78	5.35–5.91	
Gly	4.98–5.96	6.89–7.34	4.53–6.17	4.20–5.02	
Asp	7.88–9.21	7.21–8.15	7.10–10.30	9.38–9.47	
Glu	11.49–12.30	11.28–12.25	9.80–11.0	13.02–13.43	
Ala	7.40–7.65	12.29–12.73	6.57–9.17	6.58–7.95	
Pro	7.66–7.96	7.64–8.09	5.16–6.52	6.36–7.13	
Total NEAA	51.63	56.94	54.99	53.56	

^#^ Results of edible insects approved by EFSA. *Acheta domesticus* (AD); *Alphitobus diaperinus* (LMW); *Locusta migratoria* (LM); *Tenebrio molitor* larvae (TML). EAA: essential amino acid. NEAA: non-essential amino acid.

**Table 4:** Analysis of the mineral content in edible insects reared on standard diets. Results are reported in mg per 100 g of insect ^#^.

	Copper	Iron	Magnesium	Manganese	Potassium	Sodium	Zinc
AD	0.51–4.86	1.93–11.23	22.60–136.58	0.89–4.40	347–1211.10	101.44–471.4	6.71–22.20
LM	0.5–3.68	2.9–4.4	34.6–54.2	0.338–2.9	101.3–466	93.4–173.0	3.7–17.4
LMW	1.9–2.1	5.05–5.35	115–130	0.54–0.58	100–260	200–227	10–14
TML	1.6–2.0	4.6–6.7	191.6–202	0.70–1.89	726–926.8	188.4–206	6.70–12.66
Recommended daily intake (mg/day) ^a^	1200–1500	7.5–58.8	220–260	1.6–2.6	4700–5100	1200–1500	3–14

^#^ Results of edible insects approved by EFSA. *Acheta domesticus* (AD); *Alphitobus diaperinus* (LMW); *Locusta migratoria* (LM); *Tenebrio molitor* larvae (TML). ^a^ Data from FAO, WHO, and the Linus Pauling Institute.

**Table 5:** Bioactive compounds, activity, and mechanisms found in edible insects ^#^.

Bioactive Compound	Activity	Mechanism	References
Protein hydrolysates	Antioxidant	ROS quenching	[123,124]
	Antidiabetic	α-glucosidase and DPP-IV inhibition	[125,126,127]
	Antiobesity	AMPK MAPKs signalling	[128]
	Antihypertensive	ACE inhibition	[19,124]
	Antilipidemic	Inhibition of pancreatic lipase	[129,130]
	Antimicrobial	Various	[123]
Oil	Antioxidant	ROS quenching	[19,129]
Polyphenols	Antioxidant	ROS quenching	[73,131,132,133,134]
Chitosan	Antiobesity	Flocculant properties	[78]
	Antidiabetic	Enzymatic inhibition	[135]
	Antioxidant	ROS quenching	[129,130,136,137]
	Hypolipidemic	Lipid binding	[138]
	Antimicrobial	various	[139]
Bioactive peptides			
TCDSL			[120]
IDCSR			
EAEEGQF			
YAN	Antihypertensive	ACE inhibition	[122]
NICKY			[19]
QGLGY			
HILG			
NYVADGLG		α-glucosidase inhibition	
AAAPVAVAK			[140]
AR			[141]
CSR	Antidiabetic		
APVAA		DPP IV inhibition	[141]
AAGAPP			
SLVDAIGMGP AGFAGDDAPR	Antithrombotic	Interaction with thrombin 1 exosite	[142]
Tenecin 1	Antimicrobial	Gram-positive bacteria	[143]
Tenecin 2		Gram-negative bacteria	[144]
Tenecin 4			[145]
Tenecin 3		Fungi	[146]
FDPFPK VAPEEHPV	Antioxidant and anti-inflammatory	5-LOX and COX-2 inhibition	[147]
MA hydrolysateAKKHKELE	Hepatoprotective	Reduction of ROS in hepatocytes and upregulation of expression of antioxidant genes	[148]
Bioactive lipids			
MUFAs and PUFAs	Cardioprotective	Antioxidant, anti-inflammatory	[88]
			[149]
γ-tocopherol	Antioxidant	Reduction of ROS and lipid peroxidation	[150]
Phytosterols	Hypocholesterolemic	Reduction of intestinal absorption of cholesterol	[117]

^#^ Results of edible insects approved by EFSA. *Acheta domesticus* (AD); *Alphitobus diaperinus* (LMW); *Locusta migratoria* (LM); *Tenebrio molitor* larvae (TML). ACE: angiotensin-converting enzyme I, DPP IV: dipeptidyl-peptidase IV, 5-LOX: 5-lipoxygenase, COX-2: cyclooxygenase-2, MA: mealworm alcalase, ROS: reactive oxygen species.

**Table 6:** Patents and patent applications related to edible insects ^#^.

Patent Subject	Title	Publication Number	Publication Date
Rearing	Method capable of prolonging *Tenebrio molitor* larvae breeding time	CN106719456A	31 May 2017
	Breeding method capable of accelerating growth of larvae of *Tenebrio molitor*	CN106577555A	26 April 2017
	Special compound feed for adult *Tenebrio molitor*	CN110934225A	31 March 2020
	*Tenebrio molitor* breeding equipment and *Tenebrio molitor* breeding method	CN114287392A	8 April 2022
	Cleaning equipment for *Tenebrio molitor* breeding equipment	CN216369171U	26 April 2022
	*Tenebrio molitor* breeding and conveying device in sterile environment and using method of device	CN112918995A	8 June 2021
	Efficient feeding method for *Tenebrio molitor* of reproduction	CN109197787A	15 January 2019
	Breeding method of *Tenebrio molitor*	CN108849760A	23 November 2018
	Ventilation equipment for breeding *Tenebrio molitor*	CN217791081U	15 November 2022
	Breeding method for selenium-rich *Tenebrio molitor*	CN108541665A	18 September 2018
	*Tenebrio molitor* breeding and conveying device in sterile environment and using method of device	CN112918995A	8 June 2021
	Method and facility for breeding insects	US11712027B2	1 August 2023
	dsRNA as insect control agent	US9528123B2	27 December 2016
	Migratory locust rhythm genes clk, cyc, and per and application thereof in regulation and control of diapause of insects	CN108610426B	15 May 2018
	Migratory locust V-ATPase-V1 structural domain gene and application of dsRNA thereof in pest control	CN111394371B	10 July 2020
Extraction of insect components	Preparation method of *Tenebrio molitor* protein powder	CN110934221A	31 March 2020
	*Tenebrio molitor* protein extraction process	CN113563411A	29 October 2021
	Method for extracting *Tenebrio molitor* protein through superfine grinding-ultrasonic wave-microwave coupling	CN113372409A	10 September 2021
	*Tenebrio molitor* flavone and ultrasonic-assisted extraction method thereof	CN108114002A	5 June 2018
	Extraction method of *Tenebrio molitor* chitosan	CN113667036A	19 November 2021
Production of insect-based products	Production method of *Tenebrio molitor* oil	CN110938485A	31 March 2020
	Low-temperature production process of dry *Tenebrio molitor* powder	CN115254353A	1 November 2022
	*Tenebrio molitor* pupa oil microcapsule preparation method	CN106579449A	26 April 2017
	Preparation method of *Tenebrio molitor* powder rich in antibacterial peptide	CN115251236A	1 November 2022
	Preparation process for protein peptide of *Tenebrio molitor*	CN105385736A	9 March 2016
Insect Applications	Fermented mycoplasm prepared from cordyceps militaris fermented *Tenebrio molitor* and application thereof in preparation of agent for improving immunity	WO2020098397A1	22 May 2020
	Method for comprehensively utilizing *Tenebrio molitor*	CN108753433A	6 November 2018
	Method for repairing petroleum-contaminated soil	CN115415309A	2 December 2022
	*Tenebrio molitor* dung biochar as well as preparation method and application thereof	CN113860303A	31 December 2021
	A composition for recovery after surgical comprising extracts of *Tenebrio molitor*	KR20190131315A	26 November 2019
	Composition for preventing, improving, or treating sarcopenia, comprising Tenebrio molitor larval protein or hydrolysate thereof as active ingredient	US20230149475A1	18 May 2023
Applications of insects’ bioactive compounds	Method for inducing *Tenebrio molitor* to produce antibacterial peptide, preparation method of Tenebrio antibacterial peptide, and application of *Tenebrio molitor* antibacterial peptide	CN114097711A	1 March 2022
	Composition for Prevention or Treatment of Obesity Comprising *Tenebrio molitor* larva extract or *Tenebrio molitor* larva suspension	KR101651907B1	12 September 2016
	Gene for antifreeze protein derived from *Tenebrio molitor*, recombinant expression vector, engineering strain, and application of gene	CN110616227A	27 December 2019
	High-protein blood-sugar-reducing *Tenebrio molitor* biscuit and preparation method thereof	CN114847321A	5 August 2022
	Composition for Prevention or Treatment diabetes comprising *Tenebrio molitor* larva or extract suspension of *Tenebrio molitor* larva	KR101651908B1	30 August 2016
	Food composition comprising of extracts of cricket having protective effects against hepatotoxicity	KR100599460B1	6 January 2006
	Preventive and therapeutic compositions for skin inflammation comprising cricket extracts	KR20130134097A	10 December 2013
	Anticoagulant composition for inhibiting thrombin activity and food composition comprising extract of cricket fraction as effective component	KR101747245B1	4 May 2017
	Composition for preventing improving or treating thrombosis comprising extract of fermented *Tenebrio molitor* as effective component	KR102048309B1	25 November 2019
Polymer degradation	Tenebrio molitor phagostimulant and method for degrading plastic by using *Tenebrio molitor*	CN113854413A	31 December 2021
	Method for degrading waste PVC plastic film by *Tenebrio molitor*	CN115152707A	11 October 2022
	Microorganism isolated from *Tenebrio molitor* larva and having plastic degrading activity, and method for degrading plastic using same	WO2018143750A1	9 August 2018
	Breeding method of *Tenebrio molitor* capable of degrading plastic	CN108713530A	30 October 2018
	Method for rapidly degrading and cleanly utilizing waste polylactic acid by utilizing *Tenebrio molitor* larvae	CN113040096A	29 June 2021
Transportation	*Tenebrio molitor* larva living body vacuum dust-free transportation method	CN106614406A	10 May 2017
	*Tenebrio molitor* transporting and packaging structure with high survival rate	CN218704941U	24 March 2023
Insect-based food	*Tenebrio molitor* food and preparation method thereof	CN104041723A	17 September 2014
	Processing method of *Tenebrio molitor* food	CN115777923A	14 March 2023
	Production method of *Tenebrio molitor* soy sauce	CN110934283A	31 March 2020
	Formula of pet chocolate containing *Tenebrio molitor* powder and processing technology thereof	CN113841789A	28 December 2021
	*Tenebrio molitor* coarse grain bread	CN104824103A	12 August 2015
	High-protein blood-sugar-reducing *Tenebrio molitor* biscuit and preparation method thereof	CN114847321A	5 August 2022
	Yellow mealworm-chive oil cookies and preparation method thereof	CN111345337A	30 June 2020
	Breeding method for crickets comprising ginsenoside type saponin and polysaccharide from panax ginseng and their extracts and food composition	KR101716763B1	23 November 2016
	Method for preparing cricket product for food and cricket product for food prepared therefrom	KR101661176B1	25 May 2016
	Cookie and cake with insects	CN106720118A	31 May 2017
Insect-based feed	Method for supplementing *Tenebrio molitor* proteins to improve muscle quality of broiler chickens	CN109007322A	18 December 2018
	Method for preparing cricket product for food and cricket product for food prepared therefrom	KR101661176B1	2 May 2016
	Pet cat and dog food containing *Tenebrio molitor* larva zymolyte and preparation method thereof	CN112042824A	8 December 2020
Feed	Production method of high-protein *Tenebrio molitor* feed	CN114081017A	25 February 2022
	*Tenebrio molitor* adult feed	CN104814352A	5 August 2015
	Method for preparing *Tenebrio molitor* feed by utilizing rotten vegetable leaves	CN113785921A	14 December 2021
	*Tenebrio molitor* breeding feed based on crop waste and preparation method of *Tenebrio molitor* breeding feed	CN114176179A	15 March 2022
	*Tenebrio molitor* amino acid feed and preparation method thereof	CN107242382A	13 October 2017
	Method of producing *Tenebrio molitor* feed with kitchen wastes and straws, and product and apparatus	CN106173567A	7 December 2016
	*Tenebrio molitor* larva feed containing Chinese herbal medicines and preparation method thereof	CN114711348A	8 July 2022
	Degreased *Tenebrio molitor* protein feed and preparation method thereof	CN113678943A	23 November 2021
	*Tenebrio molitor* protein feed rich in various trace elements and preparation method of defatted *Tenebrio molitor* protein feed	CN111990535A	27 November 2020
Screening	*Tenebrio molitor* size and excrement classifying and screening device	CN218460097U	10 February 2023
	Screening device for separating *Tenebrio molitor* pupae, larvae, and feces	CN208879055U	21 May 2019
Cosmetics	Cosmetic or pharmaceutical composition for promoting hair growth comprising *Tenebrio molitor* fractions	KR101897720B1	12 September 2018
	Cosmetic compositions for antioxidation skin whitening or anti-inflammation comprising *Tenebrio molitor* extracts as an active ingredient	KR20180006699A	19 January 2018
	Composition containing *Gryllus bimaculatus* extract as active ingredient for improving skin wrinkles or moisturizing skin	WO2019022478A2	31 January 2019
	Cosmetic composition for skin cell regeneration	KR101720293B1	28 March 2017
	Crickets for improved antioxidant and antiaging activity, skin beauty, and cosmetic composition comprising the same	KR101809451B1	16 August 2016
	Skin external application composition for promoting wound healing or cosmetic composition for improving wrinkle comprising oil of *Tenebrio molitor* mealworm	KR20190011054A	1 February 2019

^#^ Results comprehend edible insects approved by EFSA up to December 2024, and include Acheta *domesticus* (AD); *Alphitobus diaperinus* (LMW); *Locusta migratoria* (LM); and *Tenebrio molitor* larvae (TML).

## Data Availability

No new data were created or analyzed in this study. Data sharing is not applicable to this article.

## Abbreviations

The following abbreviations are used in this manuscript:

FAO	Food and Agriculture Organization
WHO	World Health Organization
IPIFF	International Platform of Insects for Food and Feed
EFSA	European Food Safety and Authority
TML	*Tenebrio molitor* larvae
LM	*Locusta migratoria*
AD	*Acheta domesticus*
LMW	*Alphitobius diaperinus*
PUFAs	Poly unsaturated fatty acids
MUFAs	Mono unsaturated fatty acids
UFAs	Unsaturated fatty acids
EAAs	Essential amino acids
NEAA	Non-essential amino acids
ACE	Angiotensine-converting enzyme
ROS	Reactive oxygen species
DPP IV	Dipeptidyl-peptidase IV
5-LOX	5-lipoxygenase
COX-2	Cyclooxygenase-2
MA	Mealworm alcalase
UAE	Ultrasound-assisted extraction
PS	Polystyrene
PE	Polyethylene
CE	Circular economy
CAGR	Compounded average growth rate
